# Endoscopic titanium clip with nylon loop for treating esophageal and gastric variceal bleeding after endoscopic variceal ligation and transjugular intrahepatic portosystemic shunt

**DOI:** 10.1055/a-2638-5775

**Published:** 2025-08-14

**Authors:** Xubiao Nie, Yawen Li, Yongjun Liu, Changjiang Hu, Chaoqiang Fan

**Affiliations:** 1 105785 Department of Gastroenterology, The Second Affiliated Hospital of Army Medical University, Chongqing, China

A 49-year-old man experienced intermittent hematemesis and melena for over a decade. He was diagnosed with decompensated liver cirrhosis secondary to chronic hepatitis B more than 10 years ago and has since been managed conservatively with pharmacotherapy. He presented to a local hospital 8 days ago with recurrence of hematemesis and underwent endoscopic variceal ligation (EVL). Following stabilization, he was transferred to our department for a transjugular intrahepatic portosystemic shunt (TIPS) procedure.

On postoperative Day 3, the patient developed recurrent hematemesis. After fluid resuscitation and blood transfusion, an urgent gastroscopy revealed esophageal and gastric varices combined with multiple ulcers, including an ulcer of about 1 cm in diameter located on a varicose vein at the cardia with active bleeding. Re-ligation was not feasible. Therefore, we utilized titanium clips with nylon loop to suture the ulcer surface, successfully controlling the hemorrhage ( Video 1 ).

Endoscopic titanium clip and nylon loop for treating variceal bleeding after endoscopic variceal ligation and transjugular intrahepatic portosystemic shunt.Video 1

The patient’s condition improved following postoperative management, including continued fluid resuscitation and measures to reduce portal pressure, and he was subsequently discharged.

Although EVL is regarded as one of the most effective treatment modalities for esophageal varices, post-EVL bleeding remains a significant clinical challenge 1 . TIPS has been shown to be more efficacious in reducing rebleeding and rebleeding-related mortality rates in patients with cirrhotic esophageal varices 2 . Despite subsequent TIPS intervention, this patient experienced recurrent bleeding. Consequently, for patients who exhibit early bleeding following EVL combined with TIPS, the application of titanium clips with nylon loop can serve as an effective and timely remedial measure for achieving emergency hemostasis.

Endoscopy_UCTN_Code_TTT_1AO_2AD

## Notes

Endoscopy E-Videos https://eref.thieme.de/e-videos
*E-Videos* is an open access online section of the journal *Endoscopy* , reporting on interesting cases and new techniques in gastroenterological endoscopy. All papers include a high-quality video and are published with a Creative Commons CC-BY license. Endoscopy E-Videos qualify for HINARI discounts and waivers and eligibility is automatically checked during the submission process. We grant 100% waivers to articles whose corresponding authors are based in Group A countries and 50% waivers to those who are based in Group B countries as classified by Research4Life (see: https://www.research4life.org/access/eligibility/ ). This section has its own submission website at https://mc.manuscriptcentral.com/e-videos .
